# Continuous SiC Skeleton-Reinforced Reaction-Bonded Boron Carbide Composites with High Flexural Strength

**DOI:** 10.3390/ma16145153

**Published:** 2023-07-21

**Authors:** Qian Xia, Shihao Sun, Jun Ye, Cuiping Zhang, Hongqiang Ru

**Affiliations:** 1 Institute of Advanced Ceramics, School of Materials Science and Engineering, Northeastern University, Shenyang 110004, China; 1910177@stu.neu.edu.cn (Q.X.); yejun256@163.com (J.Y.); 2 Key Laboratory for Anisotropy and Texture of Materials (MOE), Northeastern University, Shenyang 110004, China

**Keywords:** reaction-bonded, boron carbide, Si infiltration, microstructures, mechanical properties

## Abstract

Reaction-bonded boron carbide (RBBC) composites have broad application prospects due to their low cost and net size sintering. The microstructure, reaction mechanism of boron carbide with molten silicon (Si), and mechanical properties have been substantially studied. However, the mechanical properties strengthening mechanism of reaction-bonded boron carbide composites are still pending question. In this study, dense boron carbide ceramics were fabricated by liquid Si infiltration of B_4_C-C preforms with dispersed carbon black (CB) as the carbon source. Polyethyleneimine (PEI) with a molecular weight of 1800 was used as the dispersant. CB powders uniformly distributed around boron carbide particles and efficiently protected them from reacting with molten Si. The uniformly distributed CB powders in situ reacted with molten Si and formed uniformly distributed SiC grains, thus forming a continuous boron carbide–SiC ceramic skeleton. Meanwhile, the Si content of the composites was reduced. Using PEI-dispersed CB powders as additional carbon source, the composites’ flexural strength, fracture toughness, and Vickers hardness reach up to 470 MPa, 4.6 MPa·m^1/2^, and 22 GPa, which were increased by 44%, 15%, and 10%, respectively. The mechanisms of mechanical properties strengthening were also discussed.

## 1. Introduction

Boron carbide ceramic has been wildly used in many industrial fields [1,2,3,4] for its outstanding physical and chemical properties [5,6]. The excellent hardness and low density make it the ideal armor material [7]. However, the dense sintering of boron carbide is extremely difficult because of its 93%-strong covalent bonds. At present, the commercial manufacturing of dense boron carbide ceramics is mainly hot pressing [8], pressureless sintering (above 2000 °C) [9], and spark plasma sintering [10]. These traditional sintering approaches place high demands on raw boron carbide purity, sintering equipment, and energy; in a word, they are costly. Meanwhile, these traditional methods are incapable of the fabrication of large-sized and complex-shaped boron carbide products.

Nevertheless, the reaction-bonded method, which was first developed in the 1970s [11], is able to prepare dense boron carbide ceramics at relatively low temperatures (1450–1600 °C) [12]. In this method, liquid Si is infiltrated into boron carbide preforms and reacts with free carbon to form SiC, and the remaining pores are filled with free Si. Manufacturing with the reaction-bonded method has distinct advantages of low-cost and pimping size change, which are important for industrial manufacturing.

Numerous research projects were conducted to improve the mechanical properties of RBBC composites. It has been shown that the reduction and refining of residual Si in RBBC composites were significantly positive to the mechanical properties [13,14,15]. By particle size distribution, free Si content, along with Si zone size, were decreased, and the Vickers hardness was improved to above 30 GPa. However, the introduction of large-sized boron carbide particles drastically decreased flexural strength and fracture toughness. The raw materials were also crucial for the RBBC ceramics’ microstructure and related characteristics. To optimize the fracture toughness, secondary phases [16,17,18] and fibers [19,20,21] were introduced into the B_4_C matrix. The secondary phases introduced into RBBC played positive roles in strengthening and toughening the composites. When carbon fibers were used as raw materials, the fracture toughness of RBBC composites reached a peak value of 7.5 MPa·m^1/2^. In recent years, carbon nanotubes [22] and polycarbosilane [23] were added to boron carbide to adjust the morphology and flexural strength of RBBC ceramics and obtained good results. By adding 1 wt.% of carbon nanotubes into RBBC composites, the flexural strength was improved to 585 MPa. As reported, the thin flattened SiC crystals at the interface of B_4_C and B-C-Si were the main reason for the improvement.

Although the previous studies have yielded many valuable results, the RBBC composites have still not been applied on a large scale. The expensive additions increased the cost greatly. If nanometer carbon black (CB) powders are adopted as additional carbon sources, the raw materials cost can be minimized, and the manufacturing process is simplified, which is beneficial to industrial manufacturing. Nevertheless, when attempting to make a mixture of B_4_C with nanometer CB powders, we found that the CB particles with nanometer sizes are difficult to disperse, and the RBBC composites prepared with untreated CB powders have violent reactions between B_4_C and molten Si, inhomogeneous microstructures, and, thus, poor mechanical properties, making them unqualified for certain applications.

In the present study, in order to fabricate RBBC composites with excellent properties and reduce the cost, nanometer CB powders were used as additional carbon sources. PEI was chosen to prepare well-dispersed B_4_C-CB powders. Dense RBBC ceramics with excellent properties were successfully fabricated with the as-prepared mixed powders. The microstructures and mechanical properties of the composites were characterized, and the strengthening mechanisms were also discussed. Meanwhile, the cost was minimized. This gives the RBBC composites a much better chance for industrial applications.

## 2. Materials and Methods

### 2.1. Raw Materials and RBBC Composites Preparation

**Figure 1:**
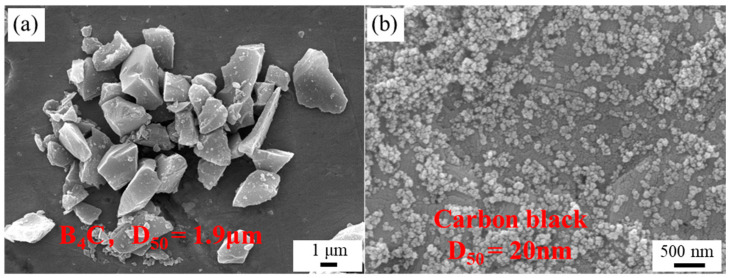
SEM images of raw material powders: (**a**) B_4_C; (**b**) CB.

B_4_C powders (China Boron Technology Co., Ltd., Weihai, China, D_50_ = 1.9 μm, Figure 1a) and nanometer CB powders (Youmeng Chemical Technology Co., Ltd., Tianjin, China, D_50_ = 22 nm, Figure 1b) were used as the starting materials. Polyethyleneimine (PEI, Aladdin Industry Co., Ltd., Shanghai, China) with a molecular weight of 1800 was chosen as the dispersant. Polyvinyl alcohol (PVA, Sinopharm Chemical Reagent Co., Ltd., Shanghai, China, hydrolysis degree of 88%) was used as the binder.

PVA and PEI were completely dissolved in deionized water. CB powders and B_4_C powders (mass ratio = 1:9) were added to the solution, and the mixture was ultrasonically dispersed for 30 min. Then, the mixed slurry was ball-milled for 24 h. After milling, the slurry was dried in the oven at 50 °C, and the obtained dry mixture was crushed and sieved through a 60-mesh sieve. For comparison, another set of mixture powder without PEI addition was also prepared. The as-prepared B_4_C-CB mixture was uniaxially pressed into bars (5 × 6.5 × 37 mm) at a pressure of 200 MPa. The compacted specimens were infiltrated with molten Si at 1600 °C for 30 min. After cooling down, the free Si on the surfaces of the infiltrated samples was treated with diamond disks, and the RBBC composites were obtained. The RBBC composites with or without PEI addition were referred to as PC-RBBC (PEI containing RBBC) and PF-RBBC (PEI-free RBBC).

### 2.2. Characterization Technique

The phase compositions of the RBBC composites were characterized by X-ray diffraction (SmartLab, Rigaku, Akishima, Japan) with CuKα radiation at 40 kV and 30 mA. 

The microstructures of the infiltrated samples were examined with a high-resolution transmission electron microscope (JEM-2100F, JEOL, Akishima, Japan) and scanning electron microscope (JSM-7001F, JEOL, Akishima, Japan). 

The open porosities and volume densities of the RBBC composites were measured via the Archimedes principle in distilled water. At least 5 samples were measured per composite (before the measurement, the composites were put into distilled water in a vacuum for 1 h, so the open pores of the composites were filled with distilled water). The open porosities and volume densities were, respectively, calculated according to the following equations:

(1) $$\rho =\frac{m_{1}}{m_{3}-m_{2}}\times \rho_{\mathrm{water}}$$

(2) $$\varepsilon =\frac{m_{3}-m_{1}}{m_{3}-m_{2}}\times 100\%$$

where *ρ* is the volume density of the composite; *m*_1_ is the dry weight of the composite; *m*_2_ is the buoyant weight of the composite in distilled water; *m*_3_ is the wet weight of the composite, and *ε* is the open porosity of the composite.

The flexural strength and the fracture toughness were tested via the three-point bending method (0.5 mm/min) and single-edge notched beam method (span = 20 mm, specimen size = 3 mm × 4 mm × 37 mm, loading speed = 0.05 mm/min) with an electronic universal testing machine (AG-XPLUS 100kN, SHIMADZU, Kyoto, Japan), respectively. At least 5 samples were tested per composite for both flexural strength and fracture toughness. The specimens for strength and toughness test were ground with a 150-grit diamond wheel and were subsequently polished with #2000 diamond disks.

The composites’ Vickers hardness was obtained via the indentation hardness method (HVS-50Z, HY, Laizhou, China), and ten independent points were measured (9.8 N, 10 s) for each sample.

For X-ray diffraction, SEM, and hardness tests, the sample was the same. The cross-sections were treated for the above tests. The section surfaces were ground with #240 and #3000 diamond disks and subsequently polished with diamond paste.

For the preparation of the TEM sample, the composites were cut into 3 mm × 0.2 mm wafers and were ground to 70 μm thickness. Then, ultra-precision dimpling grinder and ion milling were used in sequence.

The free Si of the infiltrated composites was removed via acid etching. HNO_3_ and HF were used as the etchant. Residual Si content was obtained according to the following formula:

(3) $$V_{Si}=\frac{\left(m_{1}-m_{2}\right)\cdot \rho }{2.33\cdot m_{1}}\times \%$$

where *V*_S*i*_ is the volume fraction of free S*i*; *ρ* is the volume density of the composites, and *m*_1_ and *m*_2_ are the masses of the composites before and after acid etching, respectively.

## 3. Results

### 3.1. Phase Compositions

**Figure 2:**
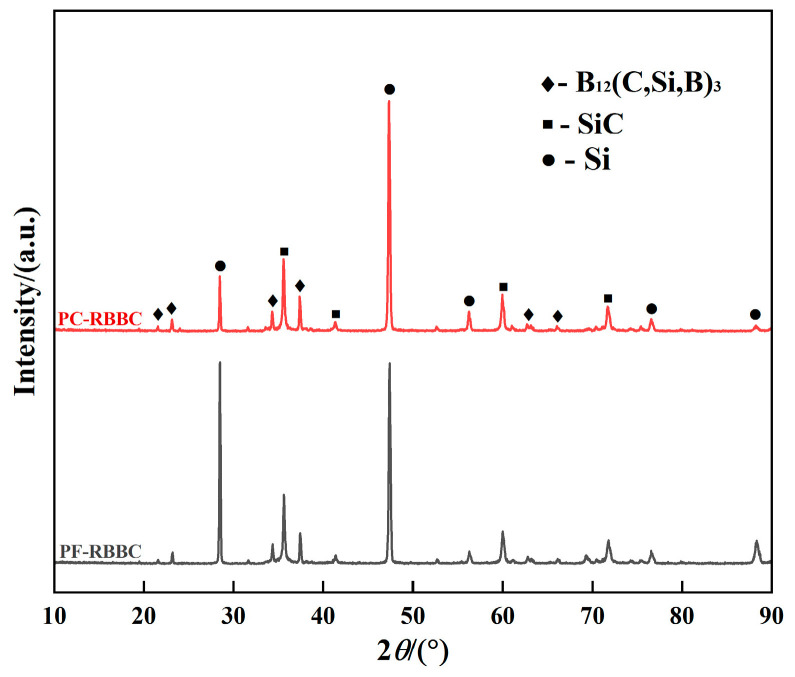
XRD patterns of different RBBC composites.

The X-ray diffraction patterns of the different RBBC composites are shown in Figure 2. Both composites consist of B_12_(C,Si,B)_3_, SiC and free Si. The addition of PEI dispersant had no obvious influence on the phase composition of the composites.

During the infiltration process, molten Si permeated into the B_4_C-CB green bodies and reacted with B_4_C and CB, and the pores of the green preforms were filled with secondary SiC and free Si; thus, the densification of the RBBC composites was achieved. The ternary phase B_12_(C,Si,B)_3_ was the product of the reaction of B_4_C with molten Si, and SiC originated from the reaction of both B_4_C and carbon with liquid Si. The corresponding equations are given below [24]:

3B_4_C(*s*) + Si(*l*)→B_12_(C,Si,B)_3_(*s*) + SiC(*s*) (4)

C(*s*) + Si(*l*)→SiC(*s*) (5)

### 3.2. Microstructure of RBBC Composites

**Figure 3:**
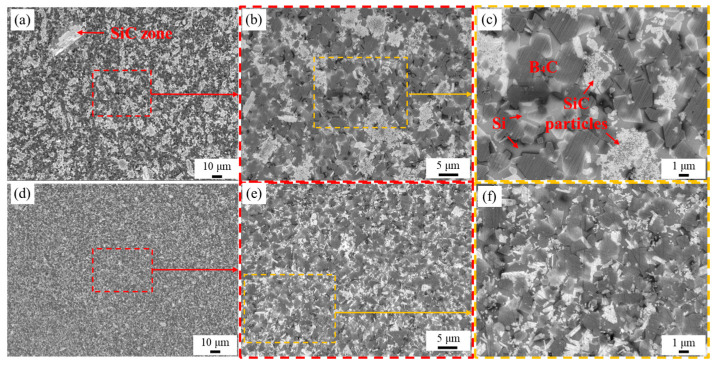
The secondary electron images of the RBBC composites: (**a**–**c**) PF-RBBC; (**d**–**f**) PC-RBBC. The SEM images were taken at progressive stages and the corresponding regions were marked by the red and yellow dashed lines.

Figure 3 shows the SEM images of the composites’ cross-sections. The dark-grey particles in the picture are the ternary B_12_(C,Si,B)_3_ phase; the continuous light-grey areas are residual Si, and the white particle is SiC. When CB powders were not dispersed, most SiC grains of the PF-RBBC composite agglomerated together, and few SiC distributed around boron carbide particles (Figure 3c). In addition, large-sized SiC grains (Figure 3a) were formed in the composite, with a size of up to 30 μm. This indicated that CB particles in green bodies were severely agglomerated. However, when PEI-dispersed CB powders were used as additional carbon sources, the microstructure of the composite was observed to be significantly uniform (Figure 3e). The SiC particles in the composite were uniformly distributed around boron carbide particles (Figure 3f), and no evidence of large-sized SiC zones was found. The SEM results fully testified that nano CB particles in the green preforms were well-dispersed when PEI was used as the dispersant; thus, the uniform distribution of the SiC grains was achieved, which originated from the dissolution–precipitation process of CB in molten Si.

**Figure 4:**
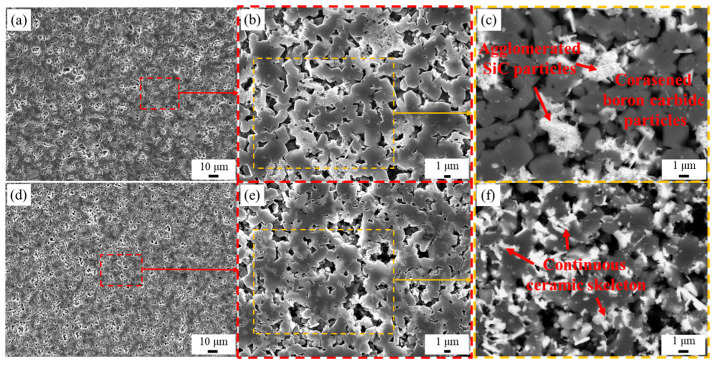
Scanning electron images of the acid-etched surfaces: (**a**–**c**) PF-RBBC; (**d**–**f**) PC-RBBC; (**c**,**f**) are the backscattered electron images corresponding to the regions in the yellow dashed line in (**b**,**e**), respectively. The SEM images were taken at progressive stages and the corresponding regions of (**b,e**) in (**a,d**) were marked by the red dashed lines.

In order to obtain a clearer view of the morphology of SiC grains in the two composites, mixed acids (HNO_3_ and HF, mass ratio = 1:1) were used to etch the free Si away, as mentioned in Section 2.2. The microstructures of the composites with residual Si were removed are shown in Figure 4.

In the composite of PF-RBBC, the SiC particles were agglomerated together (Figure 4c); as a consequence, boron carbide particles were isolated in free Si. However, in the PC-RBBC composite, which was with PEI addition, SiC particles were uniformly distributed around boron carbide particles and interconnected with boron carbide particles. In this way, a continuous three-dimensional ceramic skeleton (Figure 4f) was formed.

**Figure 5:**
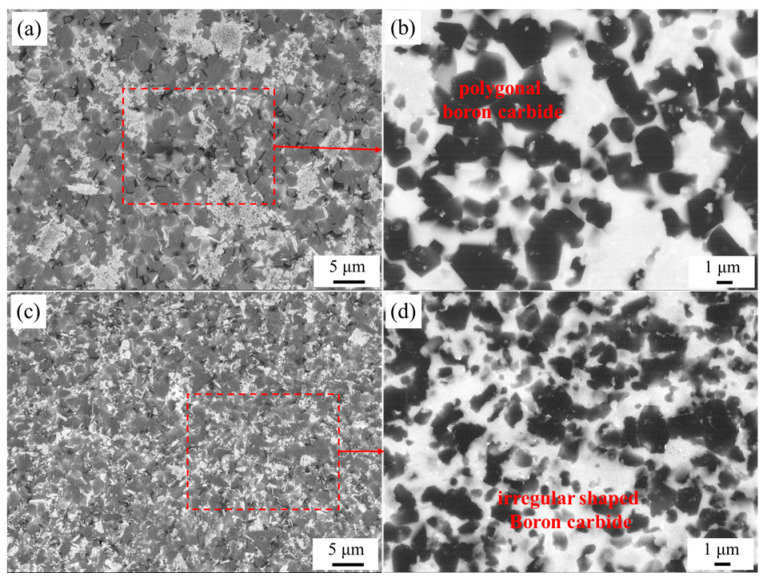
SEM images of the RBBC composites: (**a**,**b**) PF-RBBC; (**c**,**d**) PC-RBBC; (**b**,**d**) are the backscattered electron images corresponding to the regions in the red dashed line in (**a**,**c**), respectively.

Figure 5 shows the SEM images of the boron carbide particles in the two composites, in which the black particles were boron carbide. As can be seen from Figure 5b,d, boron carbide particles in PC-RBBC maintained the irregular shape of the raw material powers, while the particles in PF-RBBC were coarsened and evolved into polygons. 

**Figure 6:**
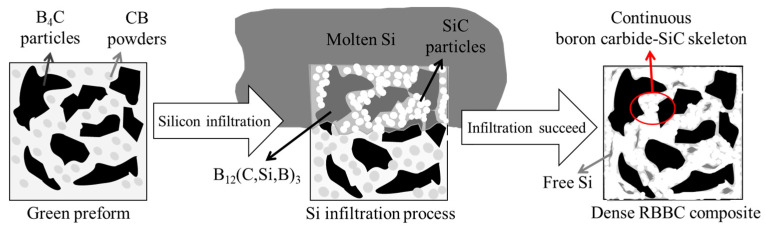
The schematic of the microstructure evolution process of PC-RBBC composite. The boron carbide–SiC skeleton was marked by the red circle.

The schematic diagram of the PC-RBBC composite’s microstructure evolution is shown in Figure 6.

The related reasons and mechanisms for the distinctions in the microstructure and morphology of the two composites are discussed as follows.

In the RBBC system, both boron carbide and free carbon react with liquid Si, as mentioned in Section 3.1. Moreover, both reactions are based on the dissolution–precipitation mechanism [25,26]: during the molten Si infiltration, raw B_4_C particles and external carbon are dissolved in molten Si; thus, the saturation of boron and carbon in liquid Si is formed. Furthermore, the ternary phase B_12_(C,Si,B)_3_ is precipitated on original B_4_C powders, while SiC is formed in molten Si at the same time [27,28]. For the PC-RBBC, CB powders were uniformly distributed around B_4_C particles in green preforms. When molten Si was infiltrated into the preforms, CB powders preferentially dissolved in molten Si, and a certain concentration of carbon was formed in the molten Si around B_4_C particles. On the one hand, the carbon concentration slowed down the rate of B_4_C dissolution in molten Si, thus indirectly protecting boron carbide from reacting with Si. On the other hand, the carbon concentration led to SiC precipitation in molten Si around boron carbide particles. As the amount of SiC increased, SiC coated one part of the boron carbide particles and served as a passivation layer at the coated regions, thus directly protecting boron carbide because of the low diffusion coefficients of Si and carbon through the SiC grains [29]. Owing to the above reasons, boron carbide particles in the PC-RBBC slightly reacted with molten Si and maintained their irregular shape. However, for the PF-RBBC, CB powders and boron carbide particles were separated from each other, and boron carbide particles were more easily dissolved in surrounding molten Si, thus leading to the violent dissolution–precipitation and the coarsening of the boron carbide grains. Meanwhile, due to the agglomeration of CB powders, SiC vastly precipitated in local regions. It is worth mentioning that the formation of SiC was an exothermic reaction [30]. The dissolution of carbon in liquid Si and the vast precipitation of SiC led to temperature increases at the reaction front [31]. The solubility of carbon and boron increased dramatically and promoted the reaction of original B_4_C and liquid Si; thus, the amount of secondary SiC increased. In the end, polygonal SiC zones were formed by the interconnection of neighboring SiC grains and their growth, as can be seen in Figure 3a.

**Figure 7:**
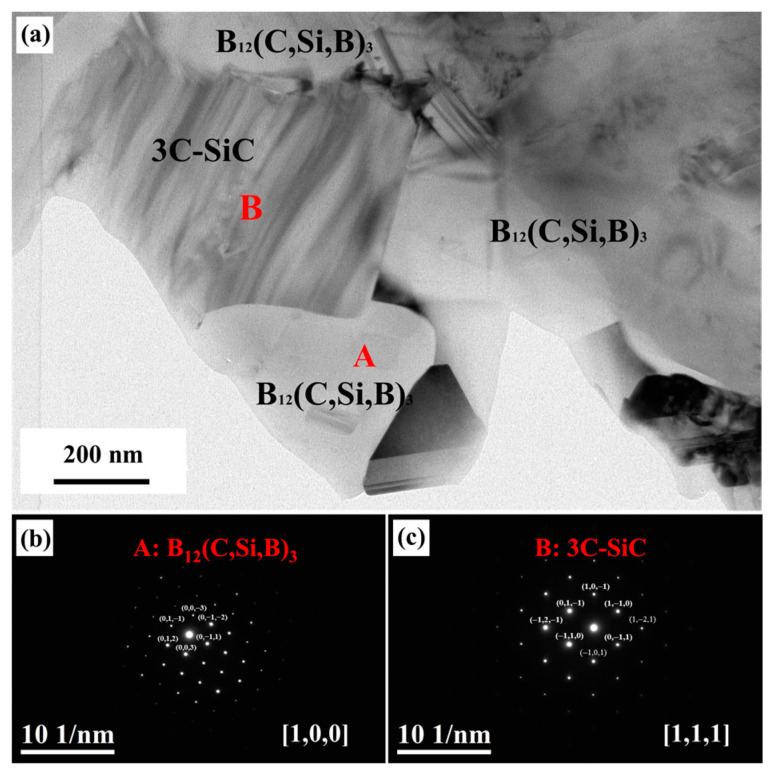
TEM images of PC-RBBC composite: (**a**) bright field image of the boron carbide–SiC skeleton; (**b**,**c**) selective area electron diffraction patterns of corresponding areas in (**a**), respectively.

The interfaces of the composite are crucial for structural integrity, load transfer, and other related properties. For the RBBC composites fabricated by molten Si infiltration, the interfaces are the reaction regions and are essential to the composites’ mechanical properties. Therefore, TEM analysis was implemented to make an in-depth understanding of the boron carbide–SiC interface. Figure 7a shows the bright field TEM image of the ceramic skeleton in the PC-RBBC composite. In the observed region, two boron carbide grains were interconnected by a SiC particle, and the interfaces of the two phases showed good bonding. The TEM analysis revealed the boron carbide–SiC ceramic skeleton with a certain strength, which will be critical to the composite’s performance.

The selective area electron diffraction patterns of region A (Figure 7b) and region B (Figure 7c) in Figure 7a confirmed the boron carbide and SiC, respectively.

**Figure 8:**
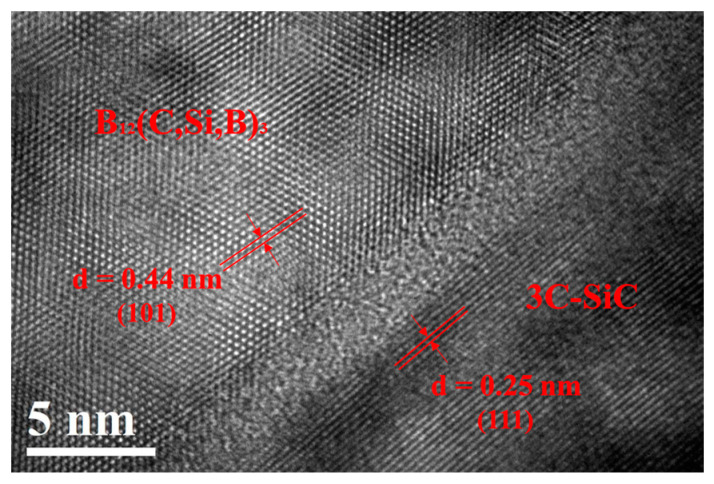
HRTEM image of the boron carbide–SiC interface of the PC-RBBC composite.

Figure 8 shows the high-resolution TEM image of the boron carbide–SiC interface. A disordered structure with a width of about 3 nm was observed at the interface, which illustrated that substantial sintering rather than simple binding was formed between boron carbide and SiC particles.

### 3.3. Density and Open Porosity

The volume densities, open porosities, and residual Si contents of the two composites are shown in Table 1. As can be seen from the table, both composites’ open porosities were below 0.4%, indicating that the two composites were dense enough. During the molten Si infiltration process, CB dissolved in liquid Si and precipitated SiC. The transition of CB to SiC led to a solid phase volume expansion of more than double [32]. The remaining pores of the green preforms were filled with liquid Si, and the densification of the composites was achieved. Due to the lower content of residual Si, the volume density of the PC-RBBC was slightly higher than that of the PF-RBBC. This indicated that the homogeneous dispersion of CB powders enabled higher packing densities and lower porosities of the pressed preforms. That is to say, by using PEI-dispersed CB as the additional carbon source, the boron carbide content of green preforms increased, and with the same amount of SiC, the pores needed to be filled with Si reduced, thus reducing the free Si content of the composites.

### 3.4. Mechanical Properties

**Figure 9:**
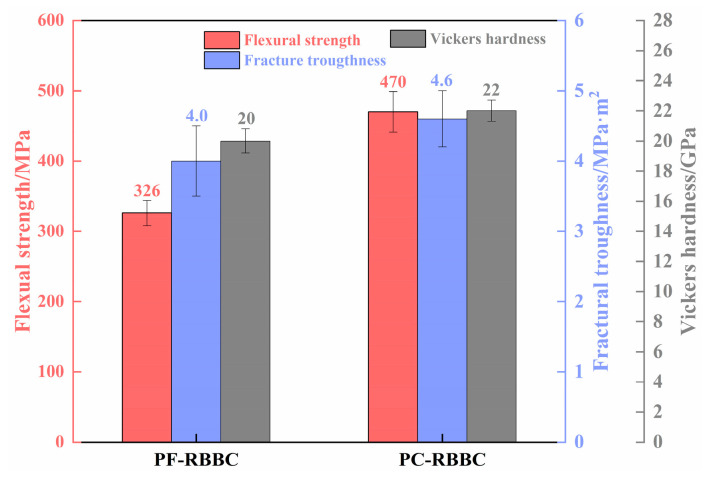
Mechanical properties of different RBBC composites.

The flexural strength, fracture toughness, and Vickers hardness of the two composites are shown in Figure 9. The flexural strength, fracture toughness, and Vickers hardness of the composites fabricated with undispersed CB powders were 326 MPa, 4.0 MPa·m^1/2^, and 20 GPa, respectively. By using PEI-dispersed CB powder as an additional carbon source, they were increased to 470 MPa, 4.6 MPa·m^1/2^, and 22 GPa, respectively. An improvement of 44% in flexural strength, 15% in fracture toughness, and 10% in Vickers hardness was achieved.

The relevant reasons and mechanisms for the enhancements in mechanical properties were analyzed as follows.

Firstly, grain size has significant effects on strength and toughness [14,33,34]. The relationship between grain size and flexural strength of ceramics has a similar relationship to that of metals, in accordance with the Hall–Petch relationship [35]:

*σ* = *σ*_0_ + *K*d^−1/2^ (6)

where *σ* is the flexural strength of the composite; *σ*_0_ is the flexural strength of the infinite single crystal; *K* is the material constant; d is the grain size of the composite. From Equation (6), it has been clear that the flexural strength decreased when the grain size increased. Boron carbide particles in PF-RBBC reacted dramatically and resulted in the size increment of boron carbide grains. Hence, the grain coarsening inevitably caused a negative impact on the flexural strength. Furthermore, research shows that the fracture toughness of the composites depends on the strain energy released during a fracture event [33]. The finer the grains in the composites, the more grains crack when extended, and the crack is more winding, thus releasing more strain energy [36]. Hence, the fracture toughness was improved. When CB powders were not dispersed with PEI, CB agglomerated together, and boron carbide particles were isolated. While molten Si was infiltrated into the preforms, boron carbide particles were directly in contacted with Si and were not protected by CB powder, thus dissolving in Si and rapidly growing. Meanwhile, large polygonal SiC regions generated in the composite also increased the average grain size. However, when PEI-dispersed CB powders were used as additional carbon sources, the uniformly distributed CB powders prevented the drastic dissolution–precipitation of original B_4_C particles in molten Si, thus avoiding the grain coarsening of the particles. Therefore, using PEI-dispersed CB powders as raw materials would prevent the grain size increase in not only boron carbide but also SiC, thereby enhancing the composites’ flexural strength, as well as the fracture toughness.

Secondly, the continuous boron carbide–SiC ceramic skeleton with well-bonded interfaces played a crucial role in the enhancements of mechanical properties [37]. When undispersed CB powders were used as an additional carbon source, the reaction-formed SiC grains and coarsening boron carbides were separated from each other, and the particles were only bonded with free Si. Free Si has much lower mechanical properties than SiC and boron carbide [38], and the flexural strength of the Si-bonded composite depended on the strength of the worst-performing phase, namely, the free Si. However, when PEI-dispersed CB powders were used as an additional carbon source, the reaction-formed SiC grains uniformly distributed around boron carbide grains and bonded boron carbide grains together, thus forming a continuous boron carbide–SiC ceramic skeleton. As can be seen in Figure 8, the atomic-level bonding of boron carbide and SiC particles greatly improved the composites’ flexural strength and fracture toughness.

In addition, the improvement in Vickers hardness benefited from the decrease in free Si content. The RBBC ceramics consist of three phases, and the hardness of different phases varies widely; the hardness of the boron carbide phase exceeds 40 GPa [39], and that of SiC and Si are reported to be approximately 20–35 GPa [40] and 9 GPa [13], respectively. For the RBBC ceramics in the present study, the composites’ hardness mainly depended on the phase contents. When CB powders were dispersed with PEI, CB powders were uniformly distributed around boron carbide particles and improved raw material powders packing density, thus improving the bulk densities and reducing the porosities of pressed green bodies. In this way, free Si content was reduced from 34.9 vol.% to 27.5 vol.% and certainly improved the Vickers hardness of the composite.

Table 2 shows the properties of similar studies in recent years, which were ordered by the flexural strength from highest to lowest. As can be seen from the table, although each property of the RBBC composites fabricated in the present study was not the best, they had no shortages. Meanwhile, the raw material (CB) used in the present study is the cheapest, and the manufacturing process is quite simple. This is important for industrialized manufacturing. The free Si content of the RBBC composites obtained in this study was high (27.5 vol.%), thus impacting the Vickers hardness. It is feasible to reduce the Si content to less than 15 vol.% to improve the composites’ hardness, which will be explored in the future.

## 4. Conclusions

In the present investigation, nanometer CB powders were dispersed using PEI by ball milling for 24 h. The PEI-dispersed CB powders were used as an additional carbon source. Dense RBBC composites were fabricated by molten Si infiltration of B_4_C-CB green preforms at 1600 °C for 30 min. The dispersed CB powders in the green preforms were uniformly distributed around B_4_C grains and efficiently protected the original B_4_C from reacting with molten Si, thus avoiding the coarsening of the particles. In the process of the molten Si infiltration, the uniformly distributed CB in situ reacted with molten Si and formed uniformly distributed SiC particles. The reaction-formed SiC particles bonded boron carbide grains together and achieved a continuous boron carbide–SiC two-phase ceramic skeleton. Meanwhile, the uniform dispersion of CB powders enabled an optimized powder stacking structure and improved the volume density of green preforms, thus reducing the free Si content of the composites. Using PEI-dispersed CB powders as additional carbon source, the flexural strength, fracture toughness, and Vickers hardness of the composites were improved to 470 MPa, 4.6 MPa·m^1/2^, and 22 GPa, which were respectively 44%, 15%, and 10% higher than those of the composites fabricated with undispersed CB powders.

## Figures and Tables

**Table 1:** Volume densities, open porosities, and residual Si content of RBBC composites.

Sample	Volume Density (g/cm^3^)	Open Porosity (%)	Residual Si (vol.%)
PC-RBBC	2.63	0.2	27.5
PF-RBBC	2.58	0.4	34.9

**Table 2:** Properties of the RBBC composites in similar recent studies.

Reference	Method	Properties			
		Flexural Strength (MPa)	Fracture Toughness (MPa·m^1/2^)	Vickers Hardness (GPa)	Volume Density (g/cm^3^)
[22]-2021	Carbon nanotubes addition	585	-	-	2.58
[21]-2019	Carbon fiber addition	510 ± 27	-	-	2.72
present study	PEI disperse CB	470 ± 29	4.6 ± 0.4	22 ± 0.7	2.63
[37]-2022	Carbon coated on boron carbide	457	4.8	24	2.576
[41]-2022	Various carbon addition	444	2.1	20.34	2.63
[15]-2019	Gel casting	419 ± 17	4.12 ± 0.12	-	2.52
[23]-2017	Polycarbosilane addition	319 ± 12	4.35 ± 0.05	17.3 ± 0.2	2.539
[42]-2021	Gel casting	299 ± 17	4.09 ± 0.17	21 ± 3	-
[24]-2018	Tape casting	245 ± 8	6.5 ± 0.5	20.2 ± 0.2	-

## Data Availability

Not applicable.

## References

[1] Wood C., Emin D., Gray P.E. (1985). Thermal conductivity behavior of boron carbides. Phys. Rev. B.

[2] Misra A.K., Greenbauer-Seng L.A. (2013). Aerospace propulsion and power materials and structures research at NASA glenn research center. J. Aerospace. Eng..

[3] Yue X.Y., Huo M.D., Liu J.Q., Wang J.J., Ru H.Q. (2022). Microstructure and properties of bilayered B_4_C-based ceramics. J. Eur. Ceram. Soc..

[4] Savio S.G., Rao A.S., Reddy P.R.S., Madhu V. (2019). Microstructure and ballistic performance of hot pressed & reaction bonded boron carbides against an armour piercing projectile. Adv. Appl. Ceram..

[5] Avcioglu S., Kaya F., Kaya C. (2020). Effect of elemental nano boron on the transformation and morphology of boron carbide (B_4_C) powders synthesized from polymeric precursors. Ceram. Int..

[6] Guo W.C., Wang A.Y., He Q.L., Tian T., Liu C., Hu L.X., Shi Y.W., Liu L.S., Wang W.M., Fu Z.Y. (2021). Microstructure and mechanical properties of B_4_C -TiB_2_ ceramic composites prepared via a two-step method. J. Eur. Ceram. Soc..

[7] Vargas-Gonzalez L., Speyer R.F., Campbell J. (2010). Flexural strength, fracture toughness, and hardness of silicon carbide and boron carbide armor ceramics. Int. J. Appl. Ceram. Technol..

[8] Mei Z., Zhou Z.H., Yuan T.C., Li R.D., Zhang W.S., Zhang Y.J., Wang M.B., Xie S.Y. (2020). Analysis of abnormal grain growth behavior during hot-press sintering of boron carbide. Ceram. Int. B.

[9] Neuman E.W., Hilmas G.E., Fahrenholtz W.G. (2022). Pressureless sintering of zirconium diboride with carbon and boron carbide nanopowder. Ceram. Int..

[10] Üsal H., Grasso S., Kovalíková A., Hanzel O., Tatarkova M., Dlouhý I., Tatarko P. (2021). In-situ graphene platelets formation and its suppression during reactive spark plasma sintering of boron carbide/titanium diboride composites. J. Eur. Ceram. Soc..

[11] Taylor K.M., Pallick R.J. (1973). Dense Carbide Composite for Armor and Abrasives. U.S. Patent.

[12] Dariel M.P., Frage N. (2012). Reaction bonded boron carbide: Recent developments. Adv. Appl. Ceram..

[13] Hayun S., Weizmann A., Dariel M.P., Frage N. (2009). The effect of particle size distribution on the microstructure and the mechanical properties of boron carbide-based reaction-bonded composites. Int. J. Appl. Ceram. Technol..

[14] Xia Q., Sun S.H., Zhao Y.L., Zhang C.P., Ru H.Q., Wang W., Yue X.Y. (2022). Effect of boron carbide particle size distribution on the microstructure and properties of reaction bonded boron carbide ceramic composites by silicon infiltration. J. Inorg. Mater..

[15] Ren Q.X., Feng D., Ru H.Q., Jiang Y., Ye C.C., Zhang Y., Wang W., Zhang C.P. (2019). Controllability of the pore characteristics of B_4_C/C preform prepared by gel-casting and the properties of RBBC composites. Ceram. Int. B.

[16] Lin Q.Q., Dong S.M., He P., Zhou H.J., Hu J.B. (2015). Microstructure and property of TiB_2_ reinforced reaction-bonded B_4_C composites. J. Inorg. Mater..

[17] Wang J.L., Lin W.S., Jiang Z.W., Duan L.H., Yang G.L. (2014). The preparation and properties of SiCw/B_4_C composites infiltrated with molten silicon. Ceram. Int..

[18] Cafri M., Malka A., Dilman H., Dariel M.P., Frage N. (2014). Reaction-bonded boron carbide/magnesium-silicon composites. Int. J. Appl. Ceram. Technol..

[19] Alexander R., Ravikanth K.V., Bedse R.D., Murthy T.S.R.C., Dasgupta K. (2019). Effect of carbon fiber on the tribo-mechanical properties of boron carbide: Comparison with carbon nanotube reinforcement. Int. J. Refract. Met. Hard Mater..

[20] Song S., Bao C., Wang B. (2016). Effect of the addition of carbon fibres on the microstructure and mechanical properties of reaction bonded B_4_C/SiC composites. J. Eur. Ceram. Soc..

[21] Solodkyi I., Bezdorozhev O., Vterkovskiy M., Bogomol I., Bolbut V., Krügerb M., Badica P., Lobod P. (2019). Addition of carbon fibers into B_4_C infiltrated with molten silicon. Ceram. Int..

[22] Karagedov G.R., Shutilov R.A., Kolesov B.A., Kuznetsov V.L. (2021). The effect of carbon nanotubes introduction on the mechanical properties of reaction bonded boron carbide ceramics. J. Eur. Ceram. Soc..

[23] Zhou Y.C., Ni D.W., Kan Y.M., He P., Dong S.M., Zhang X.Y. (2017). Microstructure and mechanical properties of reaction bonded B_4_C-SiC composites: The effect of polycarbosilane addition. Ceram. Int..

[24] Sun M.Y., Bai Y.H., Li M.X., Fan S.W., Cheng L.F. (2019). In situ toughened two-phase B_12_(C,Si,B)_3_-SiC ceramics fabricated via liquid silicon infiltration. J. Am. Ceram. Soc..

[25] Hayun S., Weizmann A., Dilman H., Dariel M.P., Frage N. (2009). Rim region growth and its composition in reaction bonded boron carbide composites with core-rim structure. J. Phys. Conf. Ser..

[26] Zhang C.P., Ru H.Q., Zong H., Sun W.K., Zhu J.H., Wang W., Yue X.Y. (2016). Coarsening of boron carbide grains during the infiltration of porous boron carbide preforms by molten silicon. Ceram. Int..

[27] Hayun S., Frage N., Dariel M.P. (2006). The morphology of ceramic phases in BxC-SiC-Si infiltrated composites. J. Solid State Chem..

[28] Hayun S., Weizmann A., Dariel M.P., Frage N. (2010). Microstructural evolution during the infiltration of boron carbide with molten silicon. J. Eur. Ceram. Soc..

[29] Matthey B., Höhn S., Wolfrum A.K., Mühle U., Motylenko M., Rafaja D., Michaelis A., Herrmann M. (2017). Microstructural investigation of diamond-SiC composites produced by pressureless silicon infiltration. J. Eur. Ceram. Soc..

[30] Ness J.N., Page T.F. (1986). Microstructural evolution in reaction-bonded silicon carbide. J. Mater. Sci..

[31] Herrmann M., Matthey B., Höhn S., Kinski I., Rafaja D., Michaelis A. (2012). Diamond-ceramics composites-New materials for a wide range of challenging applications. J. Eur. Ceram. Soc..

[32] Yurkov A.L., Starchenko A.M., Skidan B.S. (1989). Reaction sintering of boron carbide. Refractories.

[33] Barickn P., Jana D.C., Thiyagarajan N. (2013). Effect of particle size on the mechanical properties of reaction bonded boron carbide ceramics. Ceram. Int..

[34] Ellert T., Frage N. (2020). On the effects of particle size and preform porosity on the mechanical properties of reaction-bonded boron carbide infiltrated with Al-Si alloy at 950 °C. Ceram. Int..

[35] Lu Q., Zeng X.Q., Ding W.J. (2008). The Hall-Petch relationship. J. Light Met..

[36] Zhang X.R., Zhang Z.X., Sun Y., Xiang M.Y., Wang G.S., Bai Y.M., Mu J.B., Che H.W., Wang W.M. (2018). Preparation, micro-structure and toughening mechanism of superhard ultrafine grained boron carbide ceramics with outstanding fracture toughness. J. Alloys Compd..

[37] Zhang C.P., Xia Q., Han L.F., Zhao Y.L., Huang N., Ren Q.X., Zhang X., Ru H.Q. (2022). Fabrication of carbon-coated boron carbide particle and its role in the reaction bonding of boron carbide by silicon infiltration. J. Eur. Ceram. Soc..

[38] Hayun S., Dariel M.P., Frage N., Zaretsky E. (2010). The high-strain-rate dynamic response of boron carbide-based composites: The effect of microstructure. Acta Mater..

[39] Ghatu S., Blair R. (2014). Synthesis and Processing of Ultra High Hardness Boron Carbide. WIPO Patent.

[40] Patel M., Subrahmanyam J., Prasad V.V.B., Goyal R. (2010). Processing and characterization of B_4_C -SiC-Si-TiB_2_ composites. Mat. Sci. Eng. A.

[41] Sha W., Liu Y., Zhou Y., Huang Y., Huang Z. (2022). Effect of Carbon Content on Mechanical Properties of Boron Carbide Ceramics Composites Prepared by Reaction Sintering. Materials.

[42] Ren Q.X., Feng D., Ru H.Q., Jiang Y., Wang W., Zhang C.P. (2021). Effects of a hierarchical macroporous–mesoporous structure with a low porosity porous ceramic preform on mechanical properties and Si particle refinement of RBBC composites. Ceram. Int..

